# Face Ache

**Published:** 1882-11

**Authors:** 


### HALL'S JOURNAL OF HEALTH AND MISCELLANY.

MONTHLY.

E. H. GIBBS, A. M., M. D., EDITOR.

FOUNDED IN 1854.

### FACE ACHE.

HALF the human race perishes before its time, for the want of a little knowledge of the rules that govern health. The beginnings, the nuclei are few, from which radiates the host of diseases that afflict mankind. It is important, therefore, that every one should know what mischief may come from neglect of things, seemingly trivial. As an example, let us trace the possibilities connected with that very painful, but very common ailment, tooth-ache. The intense pain is caused either from an inflamed condition of the membrane that lines the tooth socket, the tooth being sound, or else from decay in the tooth itself, which has extended to the nerve. In either case we have inflammation of the membranes and nerves that are encased in unyielding channels of bone; hence the severe pain, followed by death and decay of the parts affected. While it would as a rule be unwise to resort to radical means to cure the trouble during the inflammatory stage, it is positively unsafe to neglect those means when the pain and irritation have subsided, for the *truce* is usually but temporary. If the decay extends to the surface of the tooth, the cavity forms a sort of safety valve for the escape of the dead matter, thus postponing or preventing more serious symptoms. But if the teeth are apparently sound, and there is neuralgia of the face, head, neck or shoulders, it is certain that the teeth are *not* sound, and that an expert dentist will find minute cavities extending from the crowns to the fangs of some of the teeth, or else ulcerative points at the extremities of the fangs themselves. The remedy of course is, to properly fill every cavity, being careful to make a minute examination so as to miss none. In all recent cases this is a radical cure. Should that course fail, it is certain the disease has extended beyond the reach of that remedy, and however sound appears the offending tooth it must be removed, and the removal of teeth must continue until they are all gone, if found necessary to check the neuralgia. There are, unfortunately, neglected cases where these methods are unavailing, and where the surgeon follows the disease to the cavities of the jaws, sawing through the bone and taking out the dead portion of the nerve; and still there are depths beyond the reach of human skill, where the sufferer writhes in pain until death comes to his relief; for a diseased tooth may be the beginning of fatal nervous diseases and of dyspepsia and blood poisoning. We trust this article will be carefully read, for it points out the cause and the remedy for a class of diseases that produce more intense suffering in the world than all other diseases combined.

